# The Emerging Role of Decellularized Plant-Based Scaffolds as a New Biomaterial

**DOI:** 10.3390/ijms222212347

**Published:** 2021-11-16

**Authors:** Ashlee F. Harris, Jerome Lacombe, Frederic Zenhausern

**Affiliations:** 1 Center for Applied NanoBioscience and Medicine, College of Medicine Phoenix, University of Arizona, 475 North 5th Street, Phoenix, AZ 85004, USA; ashleeharris@arizona.edu; 2 Department of Basic Medical Sciences, College of Medicine Phoenix, University of Arizona, 475 North 5th Street, Phoenix, AZ 85004, USA; 3 Department of Biomedical Engineering, College of Engineering, The University of Arizona, Tucson, AZ 85721, USA

**Keywords:** plant-based scaffolds, biomaterial, tissue engineering, cellulose, decellularization

## Abstract

The decellularization of plant-based biomaterials to generate tissue-engineered substitutes or in vitro cellular models has significantly increased in recent years. These vegetal tissues can be sourced from plant leaves and stems or fruits and vegetables, making them a low-cost, accessible, and sustainable resource from which to generate three-dimensional scaffolds. Each construct is distinct, representing a wide range of architectural and mechanical properties as well as innate vasculature networks. Based on the rapid rise in interest, this review aims to detail the current state of the art and presents the future challenges and perspectives of these unique biomaterials. First, we consider the different existing decellularization techniques, including chemical, detergent-free, enzymatic, and supercritical fluid approaches that are used to generate such scaffolds and examine how these protocols can be selected based on plant cellularity. We next examine strategies for cell seeding onto the plant-derived constructs and the importance of the different functionalization methods used to assist in cell adhesion and promote cell viability. Finally, we discuss how their structural features, such as inherent vasculature, porosity, morphology, and mechanical properties (i.e., stiffness, elasticity, etc.) position plant-based scaffolds as a unique biomaterial and drive their use for specific downstream applications. The main challenges in the field are presented throughout the discussion, and future directions are proposed to help improve the development and use of vegetal constructs in biomedical research.

## 1. Vegetal Scaffolds Are New Players to the Broader Field of Tissue Engineering

The field of tissue engineering (TE) combines materials science with cell biology to produce biological substitutes that restore tissue or organ function [1,2,3,4]. To be suitable for use in TE, this substitute must meet several requirements. Primarily, scaffolds must be biocompatible, so that cells can adhere and function normally [3,5]. The constructs must not generate a significant inflammatory response. Scaffolds must also be highly porous to allow for cell infiltration, remodeling, and growth, as well as for the removal of waste products. As cells produce their own extra cellular matrix, the scaffold should degrade with the pace of new tissue formation. Moreover, the mechanical properties should seek to mimic the anatomical area of consideration. It is also important for the scaffold to be tractable and cost-effective. To generate such scaffolds, the most common approaches fabricate from synthetic products, such as polyanhydrides or poly(ethylene glycol), which produce well-defined and reproducible structures, or natural compounds, such as cellulose [6], alginate [7], or silk [8], that offer ease of manipulation and possess unique mechanical strength. However, in recent years, another alternative has emerged from the decellularization of animal tissue. Where, after cellular removal, the structural and mechanical properties of the tissue’s extracellular matrix (ECM) remain mostly intact and allow for the repopulation of their ultra-structure with human cells to generate a tissue graft with similar features to an in vivo environment. However, the use of animal-derived sources for medical research comes with high economic cost, detrimental environmental impact, and controversial ethical considerations, as approximately 200 million animals are used annually, producing excessive energy consumption, carbon emissions, and laboratory waste [9,10]. Thus, continuous efforts need to be undertaken to develop novel biomaterials as alternatives. Awareness in this area is growing, as evidenced by the European Parliament’s joint motion for innovation to phase out animal use (adopted 13 September 2021) [11].

To meet this challenge, plant tissues have recently been decellularized to generate scaffolds suitable for TE. Using various treatments, cellular content is removed from the native plant material to generate an acellular, three-dimensional scaffold that maintains its structural, chemical, and mechanical cues [1,12]. This scaffold can then be repopulated with specific cells to produce tissue-engineered constructs for various biomedical applications such as a personalized tissue graft. Decellularized vegetal scaffolds exhibit many properties favorable for their use in TE. First, plant tissue is primarily built from cellulose. Comprising the robust plant cell wall, cellulose is hydrophilic strong/durable [13,14]. The use of this organic compound for biomedical applications has been well documented and includes drug delivery systems [15], bone/cartilage [16], vascular tissue [17], and wound healing [18], among others, suggesting that a cellulose-based plant scaffold could have biocompatible relevance for TE [19,20]. Another key attribute of plant-based biomaterials is their natural fluidic transport system, which resembles that of the branching mammalian vascular network [21]. Plant vessels diverge from large major veins into fine capillaries with detailed definition. These micro-vessels are challenging to reproduce using current three-dimensional (3D) printers or microfluidic technologies [22]. Nonetheless, these venous structures are innately found in plant architecture, increasing the attractiveness of the use of vascularized plants in TE. In addition, the intricate, natural morphology of plants is highly diversified, and, importantly, retained though the decellularization processing. This provides for a seemingly endless selection of available patterned constructs, each with different structural and biomechanical properties, that resemble and can adapt to host tissue [23,24]. Moreover, plant scaffolds are highly porous, offering openings of various sizes, many in the ideal TE range of approximately 50–200 µm in diameter [25,26,27]. As cellulose is not degraded naturally by the human body, it could be advantageous to use this scaffold in anatomical areas where scaffold collapse is often observed and requires reinforcement with metal wires [28,29]. In contrast, if a degradable scaffold is desired for implantation, there are several strategies discussed herein to achieve controlled degradation without the production of a toxic by-product [30,31]. Finally, as a sustainable resource, plant-derived scaffolds have the potential to reduce waste production, energy use, and pollution while saving time and promoting biodiversity. In summary, as such an accessible, renewable source, plants have many characteristics advantageous to their use as a biomaterial for TE and other biomedical applications.

Therefore, in recent years, several studies have started to use decellularized vegetal scaffolds to provide structural and biomechanical support for recellularization with mammalian cells, thus paving the way for the use of plant material for generating large (vascularized) tissue grafts [32,33,34,35]. As this unique area has expanded over the past few years [36], the purpose of this review is to discuss recent insights into the field. We first detail and provide perspective on the different approaches used for decellularization and recellularization of plant tissue. We evaluate the key advantages of vegetal material, including its natural and prefabricated vasculature, its specific architectural and mechanical properties, and we propose new ways in which it can be utilized for biological research. We discuss the disadvantages as well, such as the heterogeneity of the cellulose scaffold and leaf-to-leaf variation stemming from genetic or growth conditions. Finally, we consider challenging, unanswered questions in the field and future research priorities. To note, the plants cited in this review will be designated by their common name, but to avoid any confusion, an exhaustive list that specifies the formal scientific names is provided at the end of the manuscript.

## 2. Alternative Strategies to Current Chemical Decellularization Protocol

**Figure 1:**
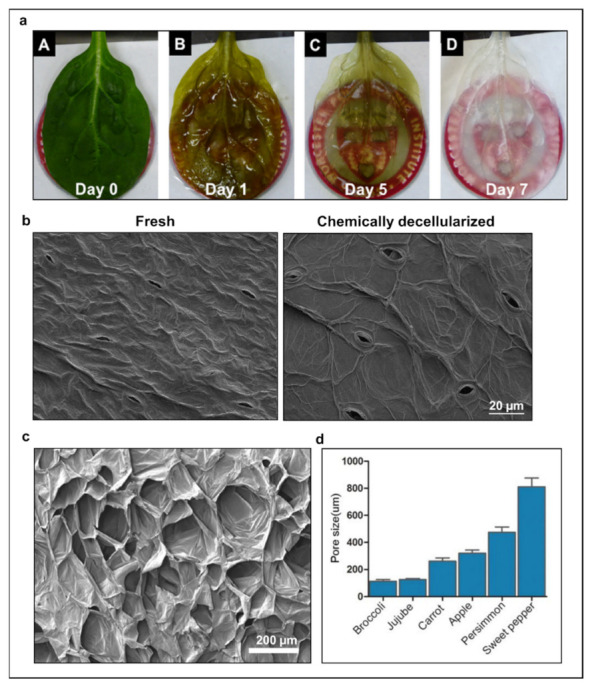
(**a**) Spinach leaf decellularization by serial chemical treatment. Perfusion of sodium dodecyl sulfate (SDS) causes the leaf to lose chlorophyll, while the bleach solution is used to remove any residual plant content and flush debris from the scaffold. Reproduced from Gershlak et al. [34]. (**b**) To visually demonstrate decellularization efficiency, scanning electron microscopy (SEM) images of fresh and chemically decellularized spinach leaf scaffolds revealed that the fullness of the fresh leaf was lost after decellularization. Cells were removed, revealing micro-vessel ultrastructure and plant features such as the cell wall and guard cells of the stomata. Data generated by authors for illustrative purposes for this review. (**c**) SEM images of scaffold architecture reveal decellularized apple tissue generates a three-dimensional scaffold. Reproduced from Modulevsky et al. [32]. (**d**) Various decellularized vegetal tissues’ pore size found in the ideal range for TE. Reproduced from Lee et al. [52].

Expertise in the decellularization of animal tissue illuminated the path forward for various potential methods of decellularization of plant material, including chemical (e.g., hypo/hypertonic solutions, detergents, solvents), physical (e.g., freeze/thaw, mechanical agitation), or enzymatic (e.g., trypsin, nucleases) approaches [26,27,37,38,39,40,41,42,43]. However, plants are robust, enduring exposure to multiple environmental elements, such as wind, rain, or sunshine, and will require stronger processing than sensitive animal tissues. Thus, chemical treatment was first investigated and has emerged, to date, to be the gold standard decellularization technique. Traditionally, chemical treatment employs an aqueous detergent (e.g., sodium dodecyl sulfate (SDS)) to solubilize cell and nuclear membranes and to denature proteins (Figure 1a,b) [1,12,44,45,46]. This is followed by a surfactant–bleach solution to clear the scaffolds of remaining debris and coloration. Chemical treatment requires intense washing post-decellularization to remove the harsh chemicals from the resulting scaffolds, as they could form a toxic residue. It should be noted that this decellularization process leaves behind a 3D scaffold whose specific architecture depends on the plant material and displays indispensable features to TE. For instance, many vegetal scaffolds were found to be highly porous (Figure 1c,d). Given the importance of pore structures for controlling cell function and for facilitating cell seeding, penetration, and distribution within the scaffold to guide the formation of new tissues or organs [47,48,49,50,51], decellularized vegetal tissues emerge as a reliable source of new biomaterial.

This chemical approach usually needs to be adapted to the composition or cellularity of the native material, specifically in length of time and chemical concentration. For instance, a low concentration of SDS was required to decellularize sensitive interior apple hypanthium [24,32,33,53] tissue, while a higher concentration and exposure time were required for more hearty material such as spinach leaves or parsley stems [34,54]. In the same way, hexane washes prior to chemical treatment can be used to remove plant wax-based cuticles where applicable.As the chemical approach utilizes strong detergent agents, this processing can often be harsh on the resulting scaffold by degrading proteins, damaging ultrastructure, and leaving behind a toxic reside [1]. Thus, optimization and alternative protocols have been explored (Table 1).

As the chemical approach utilizes strong detergent agents, this processing can often be harsh on the resulting scaffold by degrading proteins, damaging ultrastructure, and leaving behind a toxic reside [1]. Thus, optimization and alternative protocols have been explored (Table 1). For example, it has been shown that a salt treatment can remove residual SDS in the form of micelles, which then easily washes out of the scaffolds [24]. Alternatively, a second chemical approach that does not employ such detergents was shown to have similar effectiveness for decellularization [59]. Using a combination of heated bleach and sodium carbonate, the soft tissue of plant material dissolves from the plant scaffold. While this technique is based on a centuries-old approach to skeletonize leaves, and mechanical properties do not seem to be affected, it must be undertaken with caution so as to not completely degrade the scaffold.

In addition, Phan et al. demonstrated a new approach to the decellularization of plant tissue by combining physical/enzymatic means to generate a functionally biocompatible scaffold [65]. Transformed plant tissue expressing fluorescent protein (EGF) was lyophilized to permeabilize the plant cell wall prior to the use of deoxyribonuclease I (DNase I) to remove genomic content. Results showed that plant genomic material was entirely removed, whereas 36% of EFG content was preserved, suggesting that while performing an efficient decellularization, this technique can simultaneously preserve most of the protein composing the scaffold structure. However, specifically which plant proteins are needed to retain scaffold architecture or facilitate seeded human cell behavior have yet to be explored in decellularized plant scaffolds. This information would influence the decellularization method chosen and, ultimately, plant mechanical properties.

Recently, Harris et al. introduced plant material decellularization by supercritical fluid technology (i.e., compressed (supercritical) carbon dioxide, (scCO_2_)) [58]. ScCO_2_ presented an alternative option to decellularization, as the compressed carbon dioxide can penetrate dense material and act as a powerful solvent [66,67] with gas-like transport properties, liquid-like density, and lack of surface tension [68]. Decellularization of vegetal material (both tissue and stems) was achieved in the presence of a peracetic acid (PAA) co-solvent that accelerated the decellularization processing by enhancing the solubility of the scCO_2_. Similar to the chemical process, plant microarchitecture and branching vascular network were preserved in the scCO_2_ scaffolds. From start to finish, this process was shown to take approximately 36 h, as compared with the standard chemical protocol, which takes upwards of 170 h for a comparable type of material. It is of note that the authors have preliminary, unpublished data that finds the scCO_2_ treated scaffold to be possibly weakened by treatment, which requires further investigation to confirm the presence of such weakening,elucidate the possible source and optimize scCO_2_/co-solvent formula protocol.

It would be of interest for the field to have a side-by-side comparison of the different decellularization approaches on the various types of plant materials that would assess the resulting scaffolds from a physical, biochemical, and mechanical perspective. In this way, the most appropriate decellularization methods for the resulting application could be elucidated. Additionally, in order to assess these emerging approaches, there is a need to standardize the efficiency criteria for the decellularization of plant material. In 2011, Crapo et al. sagaciously proposed quantitative standards for assessing decellularization efficiency in mammalian tissues based on maintaining constructive in vivo remodeling while minimizing adverse responses [46]. The three standards are:

- *<50 ng dsDNA per mg extracellular matrix (ECM) dry weight;*
- *<200 bp DNA fragment length;*
- *Lack of visible nuclear material in tissue sections stained with DAPI or H&E.*

**Figure 2:**
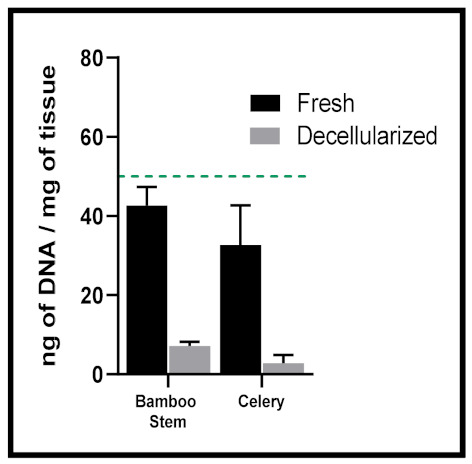
Green dashed line indicates current proposed quantitative threshold for decellularized animal tissues to be 50 ng of DNA/mg of tissue. Native plant materials such as lucky bamboo stems or celery stalks naturally fall below this level; standards should be modified to be more conducive to the extensive plant kingdom. Data generated by authors for illustrative purposes for this review.

Despite an excellent baseline and effective for animal tissue, the diverse plant community cannot always fit into such rigid categories. We have found, for example, that fresh lucky bamboo stems contain ~40 ng of DNA/mg of tissue (7.17 ng of DNA/mg of tissue after chemical decellularization) and fresh celery stalks contain ~32 ng of DNA/mg of tissue (2.86 ng of DNA/mg of tissue after chemical decellularization) (Figure 2). Moreover, plants host an important microbial community that can also be detected and lead to an overestimation when measuring DNA content from native vegetal material [69]. Standards for decellularized plant scaffolds should be adapted based on the type of tissue and plant cellularity, which would be more representative of the diverse plant kingdom.

Work in this area is in its nascent stage. Specifically, which decellularization approach is appropriate for each vegetal tissue type needs to be explored. Understanding the effects of decellularization methodology on the retention scaffold properties would be of high interest. In this way, the decellularization approach can be modified to generate an appropriate construct for the precise TE application.

To this point, advanced spectroscopy techniques have not been commonly used to evaluate or demonstrate the effectiveness of decellularization processing. However, these tools could be very useful for evaluating changes to molecules in the substrate that occurred during decellularization processing. Unfortunately, such advanced spectroscopy tools are not widely available to researchers, can be expensive, time consuming and require highly skilled users. Decellularization processing confirmation by basic staining techniques or commercially available molecular biology kits has been well established; however, it would be of great interest when establishing the most effective protocols for decellularization to employ their use to confirm architecture retention.

## 3. Decellularized Vegetal Tissues Support Cell Culture

**Figure 3:**
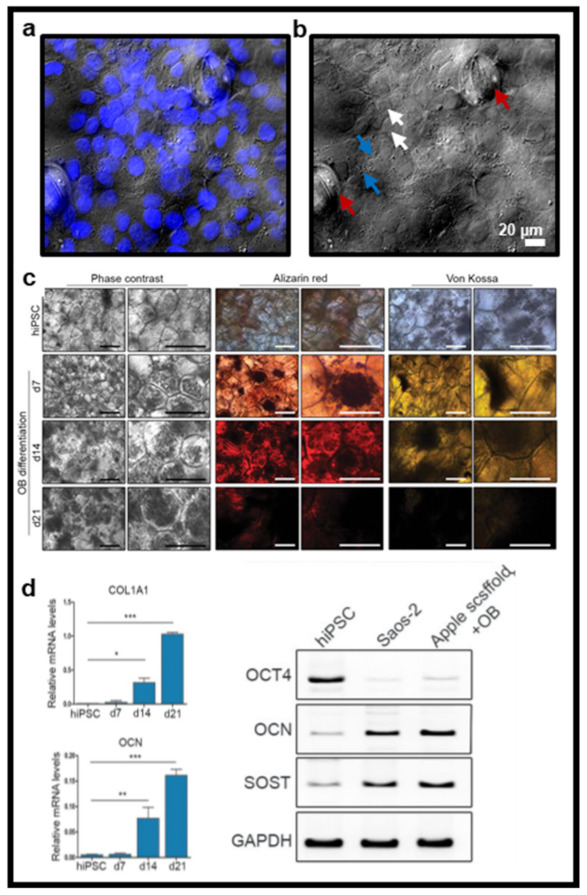
(**a**) Example of lung epithelial cells (nuclei stained with DAPI) seeded on the surface of a decellularized spinach leaf scaffold. (**b**) Brightfield image of plant scaffold alone shows plant features such as stomata (red arrow) and confirms cell attachment with the presence of cell shape imprints (white arrow) in the scaffold and points of cell attachment (blue arrow). Data generated by authors for illustrative purposes for this review using epifluorescence microscopy. (**c**,**d**) Osteoblastic differentiation of hiPSCs on 3D plant scaffold. Phase contrast images, Alizarin Red S stain and von Kossa stain before and after differentiation. Levels of osteocalcin and type I collagen mRNA expressed by hiPSCs before and after osteoblastic differentiation and expression levels of *OCT4*, *OCN*, and *SOST* mRNA after osteoblastic differentiation. Reproduced, from Lee et al. [52].

One advantage of using cellulose-based vegetal material as a scaffold is the ability to build on this tissue construct. It has been demonstrated that a variety of cell types, including human endothelial cells [34,57,62], human dermal fibroblasts (HDFs) [35,58,62,65], human skeletal myoblasts [56], human cancer cell lines [32,55,70], human aortic smooth muscle cells [64], mesenchymal stem cells [34,35,54,64], human-induced pluripotent stem cells (hiPSC) [52,63], and hiPSC-derived cardiomyocytes (hiPSC-CM) [34], as well as mouse fibroblasts [32,53,64] and mouse myoblasts [32,56] (Figure 3a,b), can attach and survive on a variety of decellularized plant scaffolds for periods of several weeks.

This was initially demonstrated on apple tissue and then subsequently shown on numerous plants and stems such as spinach leaves [34], parsley stems [35], and palm fibers [64]. Although these cells have been shown to proliferate and seem healthy for a long period, additional work is required to clearly understand how they behave on this unique biomaterial and how their metabolism and biochemical and mechanical interactions are altered if any. Interestingly, in this perspective, evidence already demonstrated that vegetal biomaterial may support stem cells differentiation (Figure 3c). For example, hiPSCs were seeded on apple-derived scaffolds and cultured in osteogenic differentiation medium for 21 days [52]. Differentiation was confirmed by the presence of mineralizing nodules and high gene expression levels of osteogenic markers osteocalcin, sclerostin, and collagen type I (Figure 3d). While this evidence alludes to a promising future for the use of vegetal-based biomaterials in TE, there are numerous species in the plant kingdom, each which requires investigation to thoroughly understand which might be most conducive to supporting cell growth/behavior.

In order to promote a sterile cell-culture environment conducive to such cell growth, plant scaffolds have been sterilized by various methods, such as UV light, ethanol, ethylene oxide (EO) gas, or penicillin/streptomycin washes [24,32,34,70]. While these are widely used techniques, the exploration of possible scaffold damage from these techniques should be investigated. For example, ethanol is drying to the scaffolds and could fracture the intricate tissue structure, while ethylene oxide (EO) has been known to deposit a toxic residue [71].

**Figure 4:**
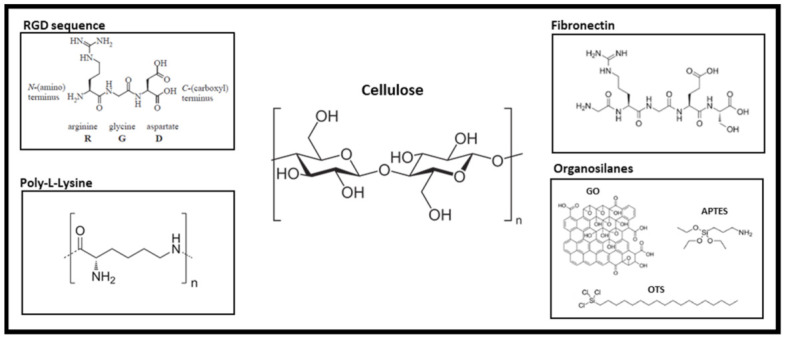
Commonly used biofunctionalization agents for promoting cell attachment to the hydroxyl groups of the cellulose-based scaffold.

Additionally, it should be noted that most of these studies employed a variety of coating types to biofunctionalize the leaf scaffold in an effort to support cell adhesion and proliferation (Figure 4). Fibronectin has been the most commonly compound used to date, either alone [34,56,63] or in combination with collagen [55,58]. Fontana et al. also investigated the use of catechol moiety conjugated peptide Arg-Gly-Asp (RGD)-Dopa to biofunctionalize the scaffold for cell adhesion [35]. The RGD sequence is the cell recognition site for attachment and is ubiquitous in adhesive proteins [72]. While the coating was found to promote cell attachment to parsley or orchid pseudobulb stems, for example, without disruption to plant topography, the drawback of this approach is that these bonds were weak and unstable when compared to a fibronectin-integrin bond as well as being non-specific, binding not just to integrins, but to other proteins as well. The authors compared the RGD-Dopa coating with a biomineralized coating. While similarly effective in promoting the expansion of human cells on the decellularized scaffolds, the latter was found to alter the topographical features of the decellularized plant stems.

Porcine skin gelatin has also been investigated for functionalization. This coating showed a stimulatory effect on cell attachment and proliferation of human dermal fibroblast cells seeded on decellularized spinach leaves for over 10 days [62]. In another study, organosilanes (3-Aminopropyl)triethoxysilane (APTES), trichloro(octadecyl)silane (OTS), and graphene oxide (GO) were compared [61]. While each promoted the viability of human neuroblastoma cells, cells seeded on the GO-coated scaffold were found to proliferate and spread more effectively. Furthermore, poly-L-lysine (PLL), a non-specific attachment factor used to promote cell adhesion, was employed to functionalized apple, celery, and carrot scaffolds prior to cell seeding and, similar to other coatings, was found to promote cell adhesion, proliferation, and differentiation [53].

Additionally, it is of note that the alkali treatment performed on palm fibers served to remove lignin and surface impurities while simultaneously adding hydroxyl groups to the scaffold [64]. This resulted in a more negatively charged, hydrophilic surface that was highly conducive to cell attachment [64,73]. Thus, surface modifications can be used to chemically cross-link scaffolds to improve coating attachment as well as cell seeding. For example, bacterial cellulose can be covalently bonded to collagen I molecules via an esterification reaction to produce stable and reproducible TE constructs [74]. Ultimately, the coating selection should be based on the cell type and the biological or clinical application of the resulting tissue construct.

Although functionalization seems to promote cell attachment and expansion on decellularized plant scaffolds, several studies have demonstrated that a coating may not be necessary for cell attachment. Various cell types have been seeded on different vegetal scaffolds that were not biofunctionalized [32,52,54,63,65,70]. For example, Robbins et al. showed that human hiPSC-CMs seeded on non-coated spinach leaf scaffolds had similar contractile function as those seeded on scaffolds coated with fibronectin or collagen IV [63].

## 4. The Exploitation of the Inherent Vegetal Vein Network to Provide a Unique Vascularized Bioengineered Tissue Construct

One of the major unmet challenges in TE is the incorporation of a functional vascular network, complete with branching generations and fine capillary detail [75]. The presence of vasculature is necessary for supporting the growing tissue by facilitating the diffusion of nutrients, gas exchange, and the elimination of waste products [76]. In addition, the tissue thickness oxygen diffusion limit is 100–200 µm, emphasizing the importance of a perfusable microvasculature (<10 µm diameter) to prevent tissue necrosis. There are many approaches to this challenge, including, bioprinting, sacrificial molding, or microfabrication [77]; yet, decellularized plant scaffolds are uniquely positioned to meet this need.

**Figure 5:**
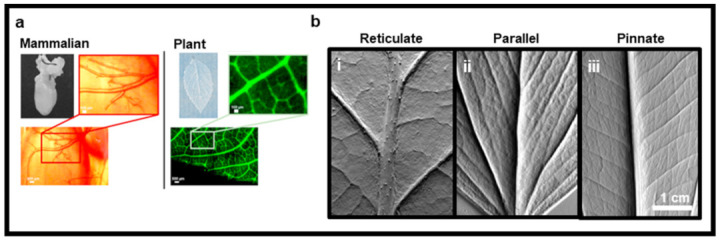
The vein network of vascular plant tissues, such as spinach leaves, tapers, and branches similar to that found in a mammalian network (**a**). Reproduced from Gershlak et al. [34]. Vascular networks of (**b-i**) spinach, (**b-ii**) lemon, and (**b-iii**) amazon sword plant leaves display various tapered patterns, including reticulate, parallel, or pinnate designs, respectively. Topographical images were obtained by authors for illustrative purposes for this review using a tactile sensor pad imaged with a GelSight, Inc., Benchtop System.

The diverse vegetal kingdom, and plant leaves in particular, have their own prefabricated vasculature. The vessels follow Murry’s Law, where they taper and branch, thus reproducing structures nearly identical to what can be observed in animal tissue [21] (Figure 5a). Interestingly, this internal and detailed architecture has been found to be preserved after decellularization. For instance, when colored dye was first perfused throughout the entirety of a decellularized spinach leaf vein network, the venous possibilities of these scaffolds were realized, as the perfusate could reach even the smallest microvessels [34]. To demonstrate the clinical potential of these results, the decellularized vascular network was shown to support the circulation of polystyrene fluorescent microspheres 1–100 µm in diameter [34]. The larger spheres (>50 µm) became stuck in the tapering vessels while the red blood cell-sized spheres (<10 µm) were able to flow through, suggesting that the branching could mimic the various dimensions of a capillary network and could even support the flow of single red blood cells (~7 µm), as observed in the smallest human capillaries. While spinach leaves represent one example of plant vasculature structure, plant material offers a large selection of vascular networks, with different designs and structures, which can be then selected according to the desired application (Figure 5b).

The natural venous network found in plant leaves has limitations. Unlike a mammalian blood vessel network, plant vasculature is not a closed loop system. Therefore, an alternative decellularized scaffold has been proposed to generate a vascularized plant construct with a flow input and output [70]. In a commanding display of control of plant tissue construction, two native plant leaves were grafted together horizontally, yet in opposite directions. As the two plants grew together into one tissue, the vessels fused via regeneration. The entire fused leaf construct was then decellularized to produce a plant scaffold with an alternative vascular flow pattern to traditional plants in which fluid, and red blood cells, can be circulated. This opened the possibility to new blood vessel connections or custom venous designs to be appropriately matched to an anatomical tissue site.

To develop a more complete cellular model, re-endothelialization of the vascular network has been attempted. However, the recellularization of these delicate biological microfluidic systems has proven to be challenging [60]. Cell injection by hand via syringe may generate a too high flow rate and lead to an increased internal pressure that would likely damage the scaffold. The use of a fluid controller (e.g., syringe pump) may help to maintain adequate flow rate and improve cell seeding efficiency; however, the important fluid dynamics and porosity of the scaffold ultimately promote cell leaking and prevent rapid cell attachment, thus leading to extremely poor seeding efficiency. This difficulty with cell adherence could also possibly be due to the presence of lignin polymers that are naturally found in vascular plant cell walls. As they provide for water transpiration over long distances, their hydrophobic nature would discourage cell attachment [78]. These organic molecules intertwine and covalently bind with cellulose, hemicellulose, and pectin to reinforce the plant cell walls; therefore, their removal from vascularized plant tissue scaffolds would likely weaken or damage the resulting scaffolds [79]. As it has been described in the previous section, biofunctionalization should be strongly considered.

To date, evidence of re-endothelization has been limited to small areas, mainly at the base of a large stem [34]. Thus, alternative approaches to cell seeding within these networks have been explored. In nature, plants use transpiration to draw water from the soil up into their vascular network, which extends from the roots into the leaves. Upon reaching the stomata (pores), water vapor is released into the atmosphere [80]. While this continuous process is no longer possible in lifeless decellularized plant tissue, the structural aspects of the transpiration network remain, as shown in a recent study [58]. Capillary tubing was inserted into the base of the stem of a decellularized spinach leaf, while the other end of the capillary tube was placed in a reservoir of ponceau red dye. The cannulated decellularized leaf was left to dry overnight at room temperature and 40% relative humidity. As the moisture evaporated from the scaffold, fluid was drawn up into the capillary tubing and through the scaffold’s venous network, reaching even the smallest capillaries. This capillary-evaporation “pump” approach could ultimately be used to draw cells deeper into the venous network or even to pre-treat the vascular network with a cell-conducive coating and before drawing endothelial cells into the venous network.

A pre-fabricated vascular template, however, is not needed to vascularize vegetal scaffolds, as shown by the subcutaneous implantation of decellularized, acellular apple tissue in mice [33]. Within one week of implantation, dermal murine capillaries had colonized the scaffold, forming new capillaries 8–25 µm in diameter. Eight weeks post-implantation, blood vessels were found extensively throughout the plant tissue, affirming the viability of the use of plant constructs for TE while also demonstrating a method by which a functional blood vessel network can be brought into an avascular plant scaffold.

In another example, pre-cellularized scaffolds were shown to be effective in promoting vascularization [62]. HDFs were seeded on a decellularized spinach leaf surface while human dermal microvascular endothelial cells were introduced into leaf vasculature. The cell-seeded constructs were then implanted into chick chorioallantoic membranes (CAM). Results showed a significant increase in the number of blood vessels that grew in the chick embryo with the pre-cellularized plant scaffolds, when compared with acellular or HDF-seeded scaffolds alone. Thus, it was concluded that scaffold pre-cellularization with vascular and supporting cells could be a promising approach for enhancing the survival of vegetal constructs in the early phases of implantation, as it could shorten the time necessary for neovascularization. This technique did not vascularize the plant material, as the vessel growth was within CAM and enhanced due to the presence of cells on the scaffold. As further studies are needed, this approach should be explored to promote the colonization of external vessels into a vegetal scaffold.

## 5. Decellularized Plant Tissues Exhibit a Wide Range of Mechanical Properties Which Can Be Matched to a Human Anatomical Site

Critical to cellular function and ultimately, tissue formation, is the result of the interaction between a cell and the surrounding microenvironment [81]. Cells in tissues can respond to mechanical stimuli (e.g., elasticity, ECM stiffness, compression of their substrate/matrix) by converting them to biochemical signals which elicit specific cellular responses in a process known as mechanotransduction. In recent years mechanosensing has been shown to be an important regulatory mechanism involved in many fundamental cellular functions such as metabolism [82], cell morphology [83], ECM homeostasis [84], tumor progression [85], etc. Thus, adequate tissue engineered scaffolds or cellular models should provide relevant biomechanical support to mimic the physiological mechanical properties of the tissue being reconstructed/simulated.

**Figure 6:**
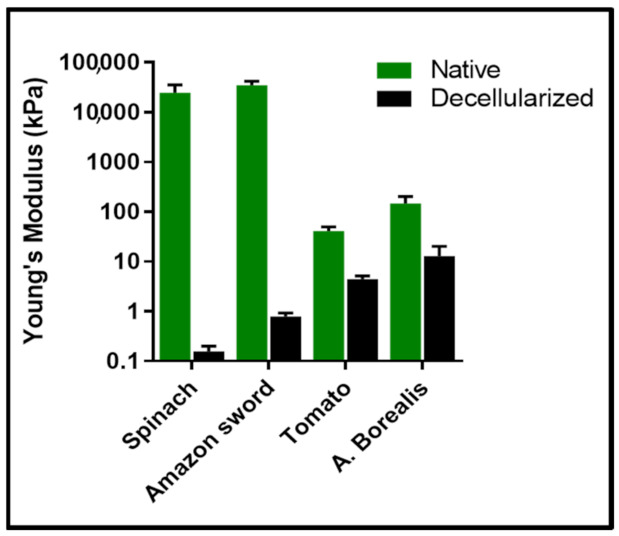
Young’s modulus of vegetal tissues before (native) and after decellularization. Graph reproduced, in part, from Lacombe et al. [55].

**Figure 7:**
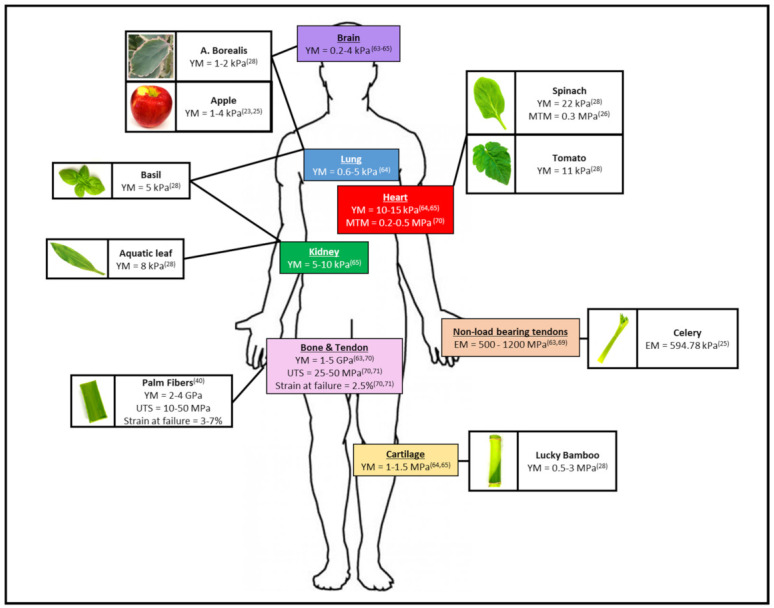
Correlation of mechanical properties between decellularized plant-based scaffold and human tissue.

One of the key advantages of vegetal material is their biologically relevant mechanical properties [86,87,88,89,90,91,92,93,94]. For example, the vegetal kingdom provides highly diversified material, such as leaves, whose stiffness can vary considerably [95]. Interestingly, many studies showed that once decellularized, the leaf stiffness, as measured by Young’s modulus (YM), decreases drastically [34,55] (Figure 6) and can reach, for some plant species, the same range of most of human tissues [31,35,53,55,61,64] such as soft organs (1–20 kPa), muscle (10 kPa), pre-calcified bone (100 kPa), or calcified cortical bone (20 GPa) [96] (Figure 7).

For example, using atomic force microscopy (AFM), the local elasticity of apple hypanthium or succulent plant leaves was found to be ~1 kPa, which is similar to that found in brain tissue. Moreover, the YM of basil and aquatic plant leaves was measured at 5.41 kPa and 8.60 kPa, respectively, and could be equated to lung or kidney tissues [32,55]. Even firmer scaffolds mirroring bone or cartilage stiffness can be recapitulated by stems such as those of the lucky bamboo plant (1.8 MPa).

Beyond stiffness, the tensile properties of decellularized vegetal scaffolds can also reach similar values to certain human tissue or organs (Figure 7). To illustrate, decellularized spinach leaves were found to have an ultimate tensile strength (UTS) of 0.05 MPa, with an 0.06 % strain at failure [34]. The maximum tangent modulus (MTM) was 0.30 MPa, which led the authors to conclude that this scaffold was similar to that of decellularized human cardiac tissue (0.20–0.50 MPa). In addition, compression testing of bulk elastic properties found celery tissue to have an elastic modulus (EM) of 594.78 kPa, similar to low-loaded anatomical tendons such as those found in the hand, while carrot tissue was shown to be in the range of non-load-bearing bone scaffolds at 43.43 kPa [53]. Despite their important influence on cellular behavior, the investigation of the mechanical properties is still understudied and only a few studies have characterized them in the resulting scaffold after decellularization. The current known data are summarized in Table 2.

Spinach leaves, and to a certain extent apple tissue, have been the most characterized plant material so far. However, because there exists a variety of mechanical testing approaches, each with different protocols and at various scales (nano vs. bulk measurement), the extraction of an absolute value or a direct comparison with human tissues should be performed with care, especially if the technique used for measurement is not clearly mentioned.

Interestingly, the mechanical properties of vegetal scaffolds can be tuned and controlled with either biofunctionalization or chemical crosslinking (Table 2). Control over the biomechanical environment is another key attribute of vegetal material. As we mentioned previously, before recellularization, vegetal tissue is often functionalized with extracellular matrix proteins, such as collagen and/or fibronectin to enhance cell adhesion. This can cause the stiffness of the functionalized scaffold to increase by a two-fold ratio: spinach leaf stiffness increased from 21.80 kPa to 37.60 kPa [55] while apple tissue was shown to increase from 0.90 kPa to 2.20 kPa [32]. Additionally, decellularized apple tissue chemically cross-linked with glutaraldehyde, a compound known to preserve and stabilize tissue, also showed an increased stiffness from 0.90 kPa to 4.10 kPa.

As biomechanical cues of vegetal decellularized scaffolds can be modified, it has also been demonstrated that cells, by their biological activity, can similarly remodel the scaffolds and alter their mechanical properties. For example, the bone-connective tissue interface region was investigated using interlocking decellularized vegetal scaffolds [97]. Two pieces, representing each region, were fit together without glue or gel to form a single unit. Each piece was repopulated with the appropriate cell type, either osteoblast cells (bone region) or fibroblast cells (connective tissue region). After 2 weeks, AFM was used to assess the local mechanical properties of each region. The bone component had been mineralized by the osteoblast cells and displayed a stiffness of 115.00 kPa, while the fibroblast cell populated region was not mineralized and had a lower modulus of 3.90 kPa. These results broaden the possibilities for decellularized plant scaffold selection and demonstrate that a scaffold can be chosen for one unmodifiable specific property (e.g., porosity) and be later tuned to match another feature (e.g., stiffness) of the desired microenvironment.

Biomaterials with matched mechanical properties will more accurately recapitulate the cellular microenvironment. To this end, preliminary investigation has demonstrated that cells seeded on soft vegetal scaffolds behave differently when compared with those seeded on the traditional, hard tissue culture plastic flasks [55,61,70]. Cancer cells grown on decellularized spinach leaf scaffolds have been shown to have downregulated YAP/TAZ signaling, decreased proliferation rates, and more rounded cell morphology than those grown on standard tissue culture flasks [55]. It was further shown that cellular response to external stress, such as drug or radiation exposure, was different between cells seeded on decellularized scaffolds and plastic flasks, highlighting the need to better characterize the cellular behavior on such scaffolds before their complete integration in multiple biomedical applications. Although evidence regarding altered cell morphology and proliferation rates on decellularized plant scaffolds has been echoed [61], other studies have shown disparate data that did not indicate a change in cell morphology or proliferation between soft vegetal and stiff plastic substrates [54,70]. Such observations were surprising since the influence of stiffness on cell behavior is well established but could be explained by the low relevance of the technical approach (visual observation instead of quantitative data) used to investigate these outcomes. Furthermore, this result could also reveal the complexity of vegetal material, as the interplay that occurs between stiffness, topography, porosity, etc. could affect the cellular phenotype in a different manner than what we observed in a simpler model. An in vivo comparison should be done to increase the relevance of all studies.

## 6. Natural Topographical Architecture Found in Plant Scaffolds Can Be Utilized to Direct Cell Behavior

Topographical cues are needed to direct organization in all tissue types—from connective to vascular tissue [98]. They have been shown to significantly influence cell behavior, such as cell adhesion, motility, shape, and ultimately, intracellular signaling pathways that regulate transcriptional activity [99]. For example, myotube formation and contractility depend on the spatial patterning of ECM proteins to direct skeletal muscle cell unidirectional alignment and differentiation [100]. Without such direction, the muscle cells will not be properly arranged and thus cannot generate contractile force. Many microfabrication approaches have been used to create these cues in bioscaffolds, such as 3D printing [101], electrospinning [102], micro-groove fabrication [103], or laser-based direct writing [104], and numerous cell types have been shown to respond to these fashioned signals. Nonetheless, such complex techniques can be expensive and time consuming to execute.

**Figure 8:**
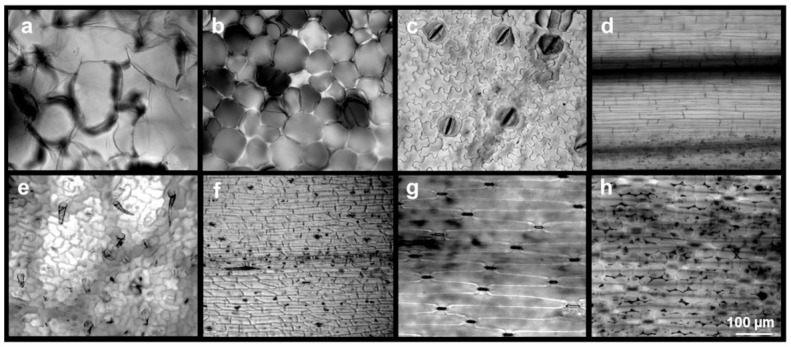
Decellularized vegetal scaffold topography. Each scaffold displays a different surface morphology, such as seen in the (**a**) apple hypanthium, (**b**) celery stalk, (**c**) aquatic plant leaf surface, (**d**) wheatgrass stem sheath, (**e**) hybrid cherry tomato plant leaf, or (**f**) curly parsley stem. Such topography can differ within a plant, as seen in the green onion’s leaf (**g**) exterior and (**h**) interior tissue. Data generated by authors for illustrative purposes for this review.

Herein again, vegetal material can overcome these limitations in tissue-engineered constructs, as they naturally display specific surface topographies that are retained through the decellularization process. Many structures, such as those found in the apple, are isotropic, appearing the same regardless of the direction of the cut of the scaffold (longitudinal or transverse) [56]. However, other materials have more anisotropic features that prominently appear in the longitudinal direction, such as the green onion (Figure 8a–h).

**Figure 9:**
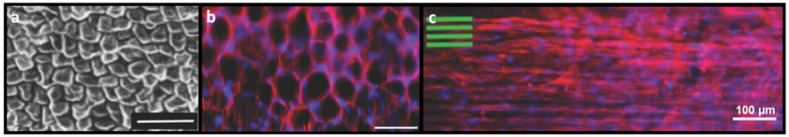
(**a**,**b**) Fibroblast cells pattern themselves around the topography of the stem of a queen anthurium. Reproduced from Fontana et al. [35]. (**c**) Cells align horizontally along pattern found in a wasabi plant stem. Reproduced from Fontana et al. [35].

Interestingly, cells seeded on such decellularized vegetal scaffolds have been shown to be responsive to topographical patterns. HDFs formed a mesh network around the microstructures of the queen anthurium stem [35] (Figure 9a,b). On a summer lilac leaf, fibroblast cells repopulated the scaffold around the leaf vasculature, as if using it as a template. Moreover, within wasabi plant stems, cells aligned in the same horizontal direction of the microstructures (Figure 8c). This alignment was quantified, and it was shown that almost 50% of the cells had an orientation angle of less than 20° (where 0° is perfect alignment). Similarly, L929 murine fibroblast cells were found to align longitudinally along the surface of decellularized celery scaffolds [53]. Moreover, 60% of the seeded cells displayed an orientation angle less than 20°, which is comparable with the aligned cell percentage on previously developed bioscaffolds replicating the tendon [70].

Cellular organization and alignment have been shown to be necessary for the formation of higher structures [105]. To explore this possibility with decellularized vegetal tissue, C2C12 myoblast cells were seeded onto decellularized green onion scaffolds [56]. Specifically, cells were seeded onto scaffolds generated from the outer portion of the green leaf and exterior portion of the white bulb. The topography of these vegetal scaffolds was found to promote uniaxial alignment and the formation into myotubes. The importance of this organization was especially stark when contrasted with the unorganized control (glass slide), where cells were randomly aligned and did not form tube-like structures.

Not all vegetal scaffolds will have appropriate microtopography. In these cases, natural, cell-secreted ECM could be a solution, as cells seeded on scaffolds will deposit their own ECM. Overall, these approaches for directing cell orientation are easy to use and more approachable than current fabrication techniques.

## 7. Biocompatibility Demonstration and the First In Vivo Applications

In order to be used as a tissue graft, vegetal scaffolds need to demonstrate low immunogenicity and biocompatibility with the animal host. As cellulose is the primary component of plant material and has been demonstrated to be biocompatible based on its use in wide-ranging medical applications [106], it was hypothesized that the plant tissue would elicit a low inflammatory response when tested for biocompatibility. Acellular, decellularized apple tissue constructs were subcutaneously implanted in murine models for periods of time ranging from 1 to 8 weeks [33]. Once removed from the host, scaffolds were evaluated and found to have retained their shape, as well as to have incited a low inflammatory profile and even to have promoted angiogenesis and ECM deposition. In a follow-up study using a similar approach but with salt-treated decellularized apple scaffolds, it was found that after 4 weeks in mice, cell infiltration was promoted to a greater extent than previously described in the first study, suggesting that the salt treatment could be used to remove the residual detergent from the decellularized scaffolds [24]. The authors hypothesized that chemicals remaining from decellularization were responsible for incomplete scaffold invasion in the first study. While likely, further investigation is needed to understand this occurrence. Biocompatibility studies were further undertaken by James et al., who assessed in vitro monocyte inflammatory gene expression and cytokine secretion in the presence of alkali treated palm fibers [64]. This natural biotextile demonstrated low immunogenicity in vivo, with mild elevation of cytokines IL-1β and TNFα.

Moreover, osteoblast-seeded apple scaffolds were grafted into rat calvarial defect models to assess bone regeneration [52]. At 8 weeks post post-engraftment, the cell-seeded scaffolds showed partial regenerative growth of the implanted area. Cells and new blood vessels of rat origin were found to have grown into the scaffolding area. Additionally, human hiPSC-derived osteoblasts were found to be present on the scaffolds, suggesting that they can survive on a vegetal scaffold inside of an in vivo system. Thus, this study underlines the potential that the appropriate scaffold type (such the porous apple) can serve as a bone graft.

In another step forward, it was recently demonstrated that decellularized plant tissue could be used as an implantable drug delivery system [107]. First, rapamycin-loaded nano-particles were conjugated onto decellularized plant scaffolds. The scaffolds were then implanted into the inferior vena cava of rats for 14 days, after which results found that the nanoparticle patches had a reduced immune profile and decreased neointimal thickness, compared with the drug-free control scaffold. While this novel application requires further examination over longer time points, it is the first to demonstrate such application with decellularized plant material and reinforces the biocompatible nature of the scaffold.

While none of the animals experienced toxicity or signs of implant rejection, low levels of macrophage cells were observed within some of the implanted scaffolds [33]. Their presence could represent very low levels of persisting inflammation or macrophage surveillance. Some of the scaffolds also showed incomplete cellular infiltration (although this was not seen in the later study). Additionally, one group of scaffolds had mild structural collapse on the edge due to animal movements. Taken together, few studies have explored the implantation of plant-based scaffolds and additional investigation is needed to assure the in vivo safety and efficacy of these tissues. As these few in vivo studies have only been observational by showing low immunogenicity and presence of host cells on the scaffold, yet without providing further explanations, deeper investigation would be required to propose a comprehensive view of the tissue formation phenomenon.

## 8. Additional Considerations for Decellularized Plant-Based Biomaterials

Beyond the optimization of decellularization/recellularization protocols and the characterization of the vegetal scaffold’s architectural and mechanical features on cellular behavior, there remain outstanding challenges to be addressed, including scaffold degradation. An ideal scaffold for implantable tissue-engineering applications would biodegrade at a controlled rate in order to keep pace with cellular remodeling and vascular infiltration [17]. However, cellulose can only be degraded by cellulase enzymes that are not present in the in vivo mammalian system [108]. Many studies have proposed options to overcome this limitation, including the codelivery of cellulase enzymes directly into the scaffold [14,32]. This has been demonstrated with bacterial cellulose (BC) scaffolds but not in plants. While the cellulose compound is the same between bacteria and plant species, in plants, the cellulose is inter-woven with other polymers, while the bacteria product is pure. In one study, BC scaffolds pre-absorbed cellulase enzymes. The scaffolds were found to degrade overtime, and the degradation rate could be controlled by modification of cellulase content to match the tissue growth rate [30]. Other proposals to this challenge have shown that pre-treating the cellulose-based scaffold with oxidizing agents can enhance cellulose degradation in vivo [31]. At the same time, it has also been suggested that scaffolds that degrade too fast can collapse before the appropriate remodeling can take place and thus could require reinforcement with permanent metal wires [28,29]. Ultimately, degradation rate should depend on specific anatomical location and tissue need—an issue that requires investigation in decellularized vegetal materials.

It has been suggested that plant-based biomaterials do not provide sufficient reproducibility, as the plants used were not grown in a controlled environment [109] where soil nutrients and age of the plant can affect structural and mechanical properties in the native leaf structures [110]. While this is a very thoughtful point, it would not be possible to make perfectly reproducible vegetal structures, as genetic and environmental factors similarly influence these properties [111]. However, with the emergence of gene editing [112], the modification of plant phenotype to obtain a more homogenous population and thus better control the inter-variation of intrinsic plant features could be an option worth exploring in order to address this specific challenge.

Importantly, vegetal-derived scaffolds hold promise as a sustainable alternative to animal-derived sources that come with high environmental impact and economic cost [9]. As “green” scaffolds, plant tissues used for TE or cellular modeling could reduce waste production, energy use, and pollution, while saving time and promoting biodiversity. There is a high demand for replacements for animal tissues for these reasons, in addition to ethical and scientific considerations, and decellularized plant materials could also be further investigated to this end. This point is further emphasized by the use of decellularized vegetal tissues as three-dimensional scaffolds on which to culture bovine skeletal muscle cells for the production of cultured meat [113,114,115,116]. In this way, the scaffolds are edible and support cell growth, while reducing costs, the need for livestock, and the occurrence of food-borne illnesses.

## 9. Conclusions

The recent advances in plant-based scaffolds have shown great potential for developing robust, low-cost cellular models that can even be built up into tissue-engineered substitutes. Once decellularized by a customized method based on cellularity, vegetal scaffolds can be biofunctionalized and seeded with human cells. As plants display a variety of stiffnesses and diverse topographies, these bio-inspired scaffolds recapitulate numerous mechanical aspects of the in vivo microenvironment that are vital for reproducing key tissue responses, as they greatly influence cell behavior. Moreover, plant leaves have an inherent vein network that can also be recellularized with endothelial cells to add a vascular component and thus more complexity than current model systems. It would be of interest to consider other applications for the intrinsic vessel network, such as for the flow of immune cells or the administration of treatments to assess drug diffusion or response. Yet, many aspects require further investigation, such as the lack of optimized decellularization protocols, limited characterization of scaffold ultra-structure post decellularization, plant-specific decellularization standards, understanding the need for biofunctionalization, one-way vascular network flow, difficulty seeding cells into the vein network, characterizing mechanical properties, effect of mechanical properties on cell behavior, role of topography in cellular organization, limited number of in vivo studies performed, scaffold biodegradability, heterogeneity of the plant material, and plant-based system reproducibility. Overall, however, decellularized vegetal tissues offer an assorted collection of complex structures with diverse biochemical and physical properties that position them as a promising and sustainable alternative biomaterial for addressing many challenges in biomedical research.

**List of plants**: Apple hypanthium (*Malus domestica*), Anthurium (*Anthurium waroqueanum*) stem, Anthurium (queen) (*Anthurium magnificum*), Amazon sword (*Echinodorus grisebachii*), Asparagus (*Asparagus officinalis*), Aurora Borealis (*Kalanchoe fedtschenkoi ariegate*), Baby sun rose (*Mesembryanthemum cordifolium*), Bamboo stem (*Bambusoideae*), Bamboo stem (*Bambusa vulgaris*), Basil (*Ocimum basilicum*), Broccoli stem (*Brassica oleracea var. italica*), Cabbage (*Brassica oleracea var. capitata*), Calathea Zebra (*Calathea zebrina*) stem, Carrot taproot (*Daucus carota subsp. sativus*), Celery stalk (*Apium graveolens*), Cucumber (*Cucumis sativus*), *Ficus hispida*, *Garcinia (species unknown*), Green onion (*Allium fistulosum*), *Impatiens capensis*, Jujube (*Ziziphus jujuba*), Leek stem (*Allium porrum*), Lucky bamboo (*Dracaena sanderiana*), Orchid pseudobulb stem (*Laelia ancepts*), *Pachira aquatica*, Parsley stem (*Petroselinum crispum*), Peanut hairy root (*Arachis hypogaea*), Persimmon (*Diospyros virginiana*), Potato (*Solanum tuberosum*), *Scheonoplectus tabernawmontani* stem, Spinach Leaf (*Spinacia oleracea*), Sweet pepper (*Capsicum annuum*), Sweet mint plant leaf (*Mentha x suavis*), Sweet wormwood leaf (*Artemisia annua*), Summer lilac (*Buddleja davidii)*, Tomato plant leaf (*Solanum lycopersicum*), Ubuçu Palm fibers (*Manicaria saccifera*), Vanilla stem (*Vanilla plainifolia*), Wasabi stem (*Solenostemon scutellarioide*).

## Figures and Tables

**Table 1:** Current protocols employed to decellularize plant material.

Decellularization Treatment	Compounds	Time	Decellularized Plants	Advantages	Limitations
**Chemical**	SDS (0.1 to 10%, depending on plant material); Triton X-100; bleach (10%) Hexane pre- treatment can be performed when wax cuticle present	12 h to 3 weeks, depending on the plant material	Amazon sword [55], Anthurium [35], Anthurium (queen) [35], Apple interior [24,32,33,53,56], Asparagus [56], Bamboo [31,35], Basil [55], Broccoli stem [52,56], Cabbage [57], Calathea zebrina [35], Carrot [52,56], Celery [53,56,58], Cucumber [56], Ficus hispida [59], Garcinia [59], Green onion [56,60], Impatiens capensis [35], Jujube [52], Leek [61], Lucky bamboo [55], Orchid pseudobulb [35], Pachira aquatica [59], Parsley stem [34,58], Peanut hairy root [34], Persimmon [52], Potato [56], Solenostemon “wasabi” [35], Spinach [34,54,55,58,60,62,63], Sweet yellow bell pepper [52], Sweet wormwood [34], summer lilac [35], tomato [55], Ubuçu Palm fibers [64], Vanilla [35]	Gold standard, well characterized; demonstrated ability to decellularize a multitude of plant materials with different structural and chemical compositions	Use of harsh chemicals; potential toxic residue thus, requires intense washing steps; time consuming; chemicals are environmentally toxic [46]
**Detergent-Free** [59]	Heated bleach and NaHCO_3_ solutions or bleach with surfactant	Minutes to hours, depending on the plant material	Bamboo stem, Ficus hispida, Garcinia, Pachira aquatica	Oxidation may enhance cellulose breakdown	Strong chemicals; able to degrade scaffold when heated [59]
**Freeze/Enzymatic** [65]	Lyophilization, DNAse I	24 h	Transgenic plant cultured cell lines: Hairy root, Tobacco bright yellow (BY-2), Monocot rice cells (*Oryza sativa* L.)	Retains native proteins	Additional clearing with surfactant might be needed to remove debris [1]
**Supercritical Fluid (scCO_2_)** [58]	scCO_2_ (2500 psi at 33 °C); PAA as cosolvent (2%); bleach if scaffold clearing required; Hexane pre- treatment can be performed when wax cuticle present	3 h (+6 h if clearing required)	Celery, Parsley stem, Spinach leaf, Sweet mint leaf	Fast; use of soft approach with minimal amount of chemicals; sterilization step included	Needs to be characterized on a larger diversity of plants; specialized equipment required [58]

**Table 2:** Summary of the mechanical properties of decellularized plant tissues. EM, elastic modulus; MS, maximum stress; MTM, maximum tangent modulus; TS, tensile strength; UTS, ultimate tensile strength; YM, Young’s modulus.

Plant	Modification	Mechanical Properties	Technique
Apple hypanthium [32,53]	None	YM = 1.10 ± 0.10 kPa	Nano-indentation
	Collagen I	YM = 2.20 ± 0.20 kPa	
	Glutaraldehyde	YM = 4.10 ± 0.30 kPa	
	Poly-L-lysine (PLL)	YM = 4.33 ± 1.98 kPa EM = 4.17 ± 0.17 kPa Residual Strain = 6.42 ± 0.08% MS = 1.17 ± 0.28 kPa	Measurement of bulk dynamic tensile properties
Amazon sword [55]	None	YM = 8.60 ± 0.70 kPa	Nano-indentation
Aurora Borealis leaf [55]	None	YM = 1.70 ± 0.30 kPa	Nano-indentation
Bamboo stem [31]	None	Compression = 1.52 ± 0.35 MPa	Measurement of bulk dynamic compression properties
	Oxidation (0.01% NaIO_4_)	Compression = 1.36 ± 0.47 MPa	
	Oxidation (0.1% NaIO_4_)	Compression = 1.08 ± 0.20 MPa	
	Oxidation (0.5% NaIO_4_)	Compression = 0.60 ± 0.05 MPa	
Basil plant leaf [55]	None	YM = 5.40 ± 2.60 kPa	Nano-indentation
Carrot taproot [53]	None	EM = 43.43 ± 5.22 kPa MS = 44.31 ± 8.59 kPa	Measurement of bulk dynamic tensile properties
Celery stalk [53]	None	EM = 594.78 ± 94.24 kPa MS = 175.93 ± 40.96 kPa	Measurement of bulk dynamic tensile properties
Ficus hispida leaf [59]	None	MTM = 2.00 MPa Strain at Failure = 0.30% UTS = 0.50 MPa	Measurement of bulk dynamic tensile properties
Leek [61]	None	EM = 4.42 ± 0.50 kPa Tensile strength = 1.89 ± 0.25 MPa	Measurement of bulk dynamic tensile properties
	APTES	EM = 1.31 ± 0.15 kPa TS = 2.45 ± 0.27 MPa	
	OTS	EM = 0.54 ± 0.14 kPa TS = 1.08 ± 0.28 MPa	
	GO	EM = 1.50 ± 0.07 kPa Tensile strength = 1.93 ± 0.10 MPa	
Lucky bamboo stem [55]	None	YM = 1.77 ± 1.20 MPa	Nano-indentation
Pachira aquatica [59]	None	MTM = 2.00 MPa Strain at Failure = 0.30% UTS = 0.50 MPa	Measurement of bulk dynamic tensile properties
Spinach leaf [34,54,55,58]	None	MTM = 0.30 MPa UTS = ~0.05 MPa Strain at Failure = ~7.00%	Measurement of bulk dynamic tensile properties
	None	Tensile testing = 1.40 MPa Strain at Failure = 4.57%	Measurement of bulk dynamic tensile properties
	None Collagen + Fibronectin	YM = 21.27 ± 0.6 kPa YM = 37.64 ± 2.3 kPa	Nano-indentation
	None (scCO_2_ treated)	YM = 18.09 ± 7.14 kPa	Nano-indentation
Tomato plant leaf [55]	None	YM = 10.70 ± 4.40 kPa	Nano-indentation
Ubuçu Palm fibers [64]	None	YM = 3.10 ± 1.04 GPa UTS = 33.96 ± 30.45 MPa Strain at Failure = 5.71 ± 2.4%	Measurement of bulk dynamic tensile properties
	Alkali treatment	YM = 8.22 ± 4.86 GPa UTS = 72.38 ± 45.19 MPa Strain at Failure = 2.80 ± 1.52%	
	Alkali treatment + autoclaved	YM = 3.10 ± 1.04 GPa UTS = 33.96 ± 30.45 MPa Strain at Failure = 5.71 ± 2.4%	

## Abbreviations

3D: three-dimensional; APTES: (3-Aminopropyl)triethoxysilane; AFM: atomic force microscopy; CAM: chorioallantoic membranes; DAPI: 4′,6-diamidino-2-phenylindole; DNA: deoxyribonucleic acid; DNase I: deoxyribonuclease I; ECM: extracellular matrix; EGF: epidermal growth factor; EM: elastic modulus; GO: graphene oxide; H&E: hematoxylin and eosin; HDF: human dermal fibroblast cells; hiPSC: human-induced pluripotent stem cells; hiPSC-CM: human-induced pluripotent stem cell-derived cardiomyocytes; IL-1β: interleukin one beta; MS: maximum stress; MTM: maximum tensile modulus ; NaHCO_3_: sodium bicarbonate; OTS: trichloro(octadecyl)silane; PAA: peracetic acid; PLL: poly-L-lysine; scCO_2_: supercritical carbon dioxide; SDS: sodium dodecyl sulfate; SEM: scanning electron microscopy; TE: tissue engineering; TNFα: tumor necrosis factor; TS: tensile strength; UTS: ultimate tensile strength; YM: Young’s modulus.
